# Realtime ultrasound guided percutaneous tracheostomy in emergency setting: the glass ceiling has been broken

**DOI:** 10.1186/s40696-017-0035-x

**Published:** 2017-11-28

**Authors:** Parli Raghavan Ravi, M. N. Vijai, Sachin Shouche

**Affiliations:** 1 5 Air Force Hospital Rowriah, Joraht, Assam 785005 India; 2 11 AFH Hindon, Ghaziabad, India; 3 MH (CTC), Pune, India

## Abstract

**Background:**

In recent years ultrasound guided percutaneous tracheostomy (USPCT) has become a routine practice in critical care units. Its safety and superiority over conventional percutaneous tracheostomy and bronchoscopic guided PCT is proven to be non-inferior in elective cases. However its role in emergency percutaneous tracheostomy has never been studied, since percutaneous tracheostomy itself remains an enigma in accessing emergency airway. There is no report of use of ultrasound guided percutaneous tracheostomy in emergency setting so far in the literature. We report our early experience with USPCT in emergency setting.

**Methods:**

Sixteen adult patients who required access to an emergency surgical airway after failure to accomplish emergency oro-tracheal intubation were the study population. Their airway was accessed by USPCT. Recorded data included clinical and demographic data including time taken to perform the procedure and complications. Short term and long term follow ups for a period of 2 years were done for the survivors.

**Results:**

Twelve male and four female patients underwent the procedure and the average time of the procedure was 3.6 min with no failures nor conversions to surgical tracheostomy and no complications. The average oxygen saturation was 86% and average Glasgow coma scale was 8.4. This time period included the oxygen insufflation time. 10 patients were decannulated while six patients died due to the pathology of the disease itself. There were no complications in either short term or long term follow up.

**Conclusion:**

USPCT has a definitive role in emergency both in trauma and non-trauma setting. It is safe, feasible and faster in experienced hands. Use of USPCT in emergency setting has further narrowed the list of contraindications of percutaneous tracheostomy.

## Background

Establishing a secure airway is a top priority in an emergency setting specially in trauma. In most circumstances, this is accomplished by endotracheal intubation. However establishing a secure airway can be difficult or impossible in a certain set of patients of head and neck pathologies, (trauma or otherwise) due to the limitations of extension of neck, upper airway edema, vomiting etc. In such cases, among the available options cricothyroidotomy is the choice. It has multiple advantages over tracheostomy being faster, safer and easier [1, 2]. Hence surgical tracheostomy is a highly unlikely preference. With the advent of percutaneous tracheostomy, the use of surgical tracheostomy has declined especially in critical care units, as multiple studies have demonstrated lesser if not equal complications with PCT against surgical tracheostomy. Traditionally, the urgent need to obtain a secure airway is considered as an absolute contraindication for percutaneous tracheostomy (PCT) [3]. The role of ultrasound in evaluation, assessment and accessing the airway has increased the practice of ultrasound guided PCT (USPCT) in most critical care units. Although a tempting alternative, USPCT has never been tried in an emergency setting, in-spite the fact that time taken to perform this procedure is as fast and easy as cricothyroidotomy. We herein report 16 cases of USPCT performed in emergency setting. We did not find any article or case report of USPCT being used to access and secure airway in an emergency.

## Methods

In the intervening period from Jan 2014 to Dec 2014, USPCT was performed in 16 patients wherein there was an urgent need to secure the airway because of the impending airway obstruction. In all cases, the emergency USPCT were done after all the modalities to secure a definitive airway by non surgical techniques was met with failure by the anesthesiologist. The non surgical techniques included supra-glottic airway support in the form of inability to bag and mask ventilation, insertion of laryngeal mask airway, i-gel and combitube. Two chances with a change of blade and one attempt with an Eschmann’s tracheal introducer (bougie) were done for achieving definitive airway with endo-tracheal intubation. All USPCTs was performed by anaesthesiologists who were trained and were doing this procedure regularly. All of the USPCTs were done on the bedside in the emergency room or in the critical care unit. The percutaneous tracheostomy technique was performed by Griggs single dilator technique. The PCT set included a puncture needle cannula complex, a guide wire, a small dilator, a curved dilator forceps tracheostomy tube. The US machine (Sonosite M turbo) was used with probe of 6–12 MHz frequency. The protocol required two operators: one dealt with the performing the PCT and another helped the operator in showing the anatomical and vascular landmarks with the ultrasound. All operators had the same level of experience and training in the use of USPCT in anesthesia and critical care medicine. The coagulation status was un-known at the time of the procedure.

**Fig. 1:**
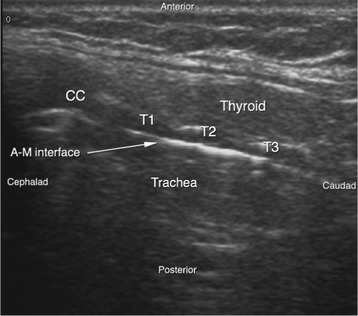
Longitudanal view of the airway. *AM* Air mucosal interface, *CC* Cricoid cartilage, *T1* Ist tracheal cartilage, *T2* 2nd Tracheal cartilage, *T3* 3rd tracheal cartilage

**Fig. 2:**
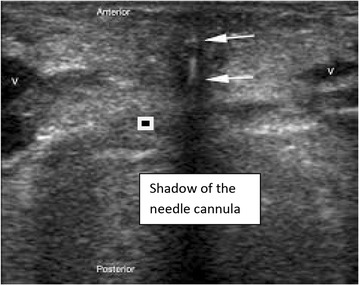
Transverse view. *V* Veins

**Fig. 3:**
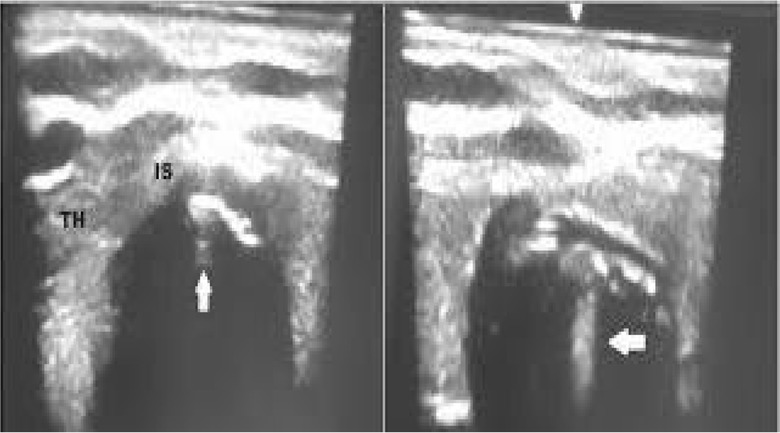
Needle cannula complex in the Tracheal lumen. *TH* Thyroid gland, *IS* ISthamus, Arrow showing the needle with cannula

Most of the patients were in the supine position with cervical collar on the spine board. The front part of the Philadelphia collar was removed when the procedure was being conducted. Continuous oxygen through the face mask was provided. Airway manevoure in form of chin lift and jaw thrust were given by the para-medical staff in majority of the patients, however it was not possible in all patients due to the distorted neck anatomy secondary to trauma. After a quick disinfection and preparation a US examination of the neck region was done in the longitudinal view to identify the cricoid cartilage, the tracheal rings, and the puncture site was decided between the first–second or second–third tracheal rings (Fig. 1) depending upon the presence of arteries, veins and thyroid isthmus in the transverse plane. The transverse plane also helped to measure the thickness of the skin to the anterior tracheal wall. A gentle rostral traction was given to appreciate the field better by the operator. Local anaesthesia with 1% lignocaine was given following which a small horizontal incision of 1 cm was made over the pre-decided puncture site. After doing some blunt dissection of the soft tissues the tracheal wall was punctured with a needle cannula under real time US guidance. The needle with cannula was identified in transverse view. The needle cannula complex appears as a shadow in the soft tissue (Fig. 2), which after entering the airspace of trachea appears as a clear white shadow (Fig. 3). The needle was removed and the cannula was connected to an oxygen source for a period of 30–45 s. This was done to improve the oxygenation in the patients. The guide wire was introduced through the cannula and was visualized as a hyperechoic signal on transversal and longitudinal sections. The small dilator was then used to create the initial stoma followed by which the single-stage Griggs forceps dilator was passed over the guide wire and dilatation of the trachea was done. The tracheostomy tube was introduced into the lumen by a rotational movement over the guide wire. The placement of the tracheostomy tube was confirmed using multiple procedures which included auscultation, verification of appropriate breath delivery on the ventilator and the presence of the sonographic “lung-sliding” bilaterally. Complications during the PCT procedure were monitored. All patients had an endoscopic check before decannulation of the patient (even in dead patients) or before ICU discharge for non-decannulated patients. All patients who survived underwent a follow up at intervals of 3 months, 6 months and 1 year.

## Results

Sixteen patients in a busy tertiary care centre underwent USPCT either in the emergency department or critical care unit. 12 of them were male and four were female patients. The average age of the patients was 32 years, with the eldest being 47 years, and the youngest being 18 years. Eight of the patients had severe maxillofacial injuries wherein the anatomical distortion and the bleeding into the oro-facial cavity made it impossible for endo-tracheal intubation (ETT). Four of them had suspected cervical cord injuries of which 02 were confirmed later by radiological studies. Only in two patients ETT was tired resulting in failure. In all the remaining patients, USPCT was directly selected as the first mode to have secure airway. Five patients were of severe head injury along with suspected cervical cord injury. ETT was tried in all these patients without success. Two patients had inhalational burns with severe airway edema, causing an inability to access the airway. One patient was morbidly obese and had a cardiac arrest and was not able to be either ventilated or intubated. All the patients except one were spontaneously breathing although the efforts were not adequate enough to maintain the oxygen saturation above 90%. The average oxygen saturation was 86% (83–89%), which improved after the cannula (92–96%) was placed and oxygen supplementation (100%) was given. The average oxygen saturation 5 min after the procedure was 98% (95–99%). Neck extension was used only for one patient. The average Glasgow coma scale (GCS) before the procedure was 8.4 (6–11). Five minutes after the procedure it was 9.2 (6–13). Seven patients were highly agitated and required sedation with fentanyl and midazolam. Placement of supra-glottic airway device was tried to be placed only in one case without success. One patient required cardio-pulmonary resuscitation before the procedure. The maximum time from the skin incision to tube placement was 4.6 min and the minimum time was 3.4 min, with an average of 3.6 min (Table 1). This time included the time for US identification of landmarks, and 30–45 s of oxygenation through the cannula. There were no procedure linked complications in the early (in the hospital) and late (till 1 year in the survivors’). Six patients died of causes unrelated to USPCT due to their primary pathology. All the other patients were de-cannulated within 6 months.

**Table 1:** Clinical and procedural data

Variable	Result
Age	32 (18–47)
Sex (male)	12
Patient details	
Maxillofacial injury	8
Head injury	5
Burns	2
Acute myocardial infarction	1
Oxygen saturation (before procedure)	86% (83–89)%
Oxygen saturation (after procedure)8	98% (95–99)%
Glasgow coma score	8.4 (6–11)
Injury severity score	26.76 (16.43–29.37)
Length of operation time (min)	3.6 (3.4–4.6)
Decanulation	10
Complications	Nil

## Discussion

Cricothyroidotomy is a procedure classically used in emergency airway situations. However this procedure has long term complications, and has to be converted to formal tracheostomy. Percutaneous tracheostomy is considered as an absolute contradiction for airway access because of acute airway compromise. However some isolated studies and case reports have reported the safety and feasibility of it in emergency airway access [4–6]. All these studies used conventional method of PCT. In the last decade the use of ultrasound for PCT has proven to be safer, faster to perform with lesser short and long term complications. In the true sense in most critical care units USPCT has become a gold standard for surgical airways when it is done as an elective process.

Davidson et al. [7] did the largest retrospective study on emergency PCT involving 18 patients. The authors described successful placement of PCT in all patients. In eight of these patients they had a temporary access to the airway with a supra-glottic device in the form of a combitube or laryngeal mask airway. None of our patients had any supra-glottic airway, device except a facemask with 100% oxygenation. This may be due to the fact the indications for PCT were different. Also in our study all the patients underwent USPCT in the emergency room or in the critical care ward and the procedure were done by anaesthesiologists who were trained in USPCT, unlike in the study of Davidson et al. wherein the procedures were done at different wards by physicians of different specialties (General surgeons, thoracic surgeons, anaesthesiologists and otolaryngologist). Most of our patients were having maxillofacial injuries with distorted airway and with bleeding into the airways. In patients with cervical cord injuries with failed intubation, we resorted immediately to USPCT as it was made available in our setup at the bedside itself. Further the time taken to perform a conventional PCT unaided has more chances of complication, and can be time consuming in comparison to USPCT since ultrasound gives a real time appreciation of the airway which can improve the confidence of the operator.

Ben-Nun et al. [8] did a retrospective study using the Griggs technique which involved 10 patients requiring PCT in emergency. All the PCTs were conventional, unaided and done by thoracic surgeons. The average time to perform PCT in the study was 5.5 min, which in our study was 3.4 min. Both the times include the oxygen insufflation time. The average time is lesser than in our study, although a prospective randomized trial comparing the conventional PCT and USPCT used should only be commented upon. Presently there is not even a case report of USPCT in literature.

Blind PCT has several disadvantages, including the failure to recognize the point of entry of the needle, the position of the tracheostomy tube, false passage, damage to the esophageous and close structures [9]. The use of bronchscopic assistance for the better demarcation of the point of entry of the needle and avoidance of damage to structure is not feasible in patients in emergency and trauma since it is time consuming and requires patient cooperation. Moreover if such access is available the chances of obtaining definitive airway by conventional intubation in trauma or emergency would have been feasible. It may be a good alternative for PCT if the patient is already intubated, although studies are there which may contest the rate of complication and ease of doing the procedure, in comparison to USPCT even in non-emergent situations [10–13].

USPCT has numerous advantages. It facilitates the clinician towards identifying the vascular structures, thyroid, delineating the airway, evaluating the thickness of the skin over the neck and visualizes the needle and guide wire passage [14]. The incidence of early complication of bleeding hypotension, pneumothorax, puemomediastinum, failure of procedure, damage to the posterior wall and late complications of tracheal stenosis, tracheomalacia trachea-esophageal fistulae and others are less in comparison to conventional blind PCT [10, 11].

In our study, we report a very positive experience with the USPCT and found it to be safe and feasible. Actually, in an emergency setting USPCT is easier when compared to conventional tracheostomy, and conventional blind PCT. A comparative study with the gold standard cricothyroidotomy, will be ideal to establish its practice. Although in Davidson et al. study they did PCT in two patients who had failed cricothyroidotomy, in all our patients the indication was an impending catastrophic airway obstruction secondary to failure of ETT or the inability to do it. All the patients except one were breathing, (although dyspenic) and thus USPCT was preferred over complication prone cricothyroidotomy. We gave oxygen insufflations to all patients through cannula, which is equivalent to cricothyroidotomy. So in reality we did two procedures in the same sitting without any complications of either procedure, without much difficulty. Studies have reported that in experienced hands the USPCT was good alternative if not equal to BPCT in critical care unit in patients undergoing PCT [11]. In this study we found that UPSCT done in experienced hand is a safe, easy and fast, life saving procedure in emergency situations especially in trauma.

With the use of ultrasound the contraindications of PCT are coming down day by day. It’s being contraindicated in emergency and trauma has been questioned by many authors and with use of ultrasound for the access of airway a thought to remove it from the list should be seriously considered. However larger, randomized studies are required to better define the relative advantages of this technique, appropriate candidates, the safety and long term complications of US-PCT in emergency and trauma. For other teams to complement our results with little to no training in US or cervical anatomy may be difficult, as the required expertise may not be there and this is largely only a small study to confirm the superiority of USPCT over conventional way to achieve surgical airway. However the option appears to be very attractive.

## Conclusions

US-guided PCT can be performed safely by experienced physicians in emergency setting both in trauma and non-trauma situations. Use of US gives a better understanding of the anatomy of the neck and prevents vascular puncture and other complications.

### Limitations

There were certain limitations to the study. Firstly, the sample size was too less, it cannot conclude that it is a better in comparison to the gold standard (surgical cricothyroidotomy) needs to be proved in a large scale randomized controlled trial. The other limitation was that there are only two anaesthesiologist trained in doing USPCT in emergency; hence all patients did not have the access to it, which is an obvious bias in the study.
